# Development and validation of a prediction model using molecular marker for long‐term survival in unresectable stage III non‐small cell lung cancer treated with chemoradiotherapy

**DOI:** 10.1111/1759-7714.14218

**Published:** 2021-12-19

**Authors:** Yufan Yang, Tao Zhang, Zongmei Zhou, Jun Liang, Dongfu Chen, Qinfu Feng, Zefen Xiao, Zhouguang Hui, Jima Lv, Lei Deng, Xin Wang, Wenqing Wang, Jianyang Wang, Wenyang Liu, Yirui Zhai, Jie Wang, Nan Bi, Luhua Wang

**Affiliations:** ^1^ Department of Radiation Oncology, National Cancer Center/National Clinical Research Center for Cancer/Cancer Hospital Chinese Academy of Medical Sciences and Peking Union Medical College Beijing China; ^2^ State Key Laboratory of Molecular Oncology, Department of Medical Oncology, National Cancer Center/Cancer Hospital Chinese Academy of Medical Sciences & Peking Union Medical College Beijing China; ^3^ Department of Radiation Oncology, National Cancer Center/Cancer Hospital & Shenzhen Hospital Chinese Academy of Medical Sciences and Peking Union Medical College Shenzhen China

**Keywords:** chemoradiotherapy, nomogram, non‐small cell lung cancer, survival

## Abstract

**Background:**

This study aimed to establish a predictive nomogram integrating epidermal growth factor receptor (*EGFR*) mutation status for 3‐ and 5‐year overall survival (OS) in unresectable/inoperable stage III non‐small cell lung cancer (NSCLC) treated with definitive chemoradiotherapy.

**Methods:**

A total of 533 stage III NSCLC patients receiving chemoradiotherapy from 2013 to 2017 in our institution were included and divided into training and testing sets (2:1). Significant factors impacting OS were identified in the training set and integrated into the nomogram based on Cox proportional hazards regression. The model was subject to bootstrap internal validation and external validation within the testing set and an independent cohort from a phase III trial. The accuracy and discriminative capacity of the model were examined by calibration plots, C‐indexes and risk stratifications.

**Results:**

The final multivariate model incorporated sex, smoking history, histology (including *EGFR* mutation status), TNM stage, planning target volume, chemotherapy sequence and radiation pneumonitis grade. The bootstrapped C‐indexes in the training set were 0.688, 0.710 for the 3‐ and 5‐year OS. For external validation, C‐indexes for 3‐ and 5‐year OS were 0.717, 0.720 in the testing set and 0.744, 0.699 in the external testing cohort, respectively. The calibration plots presented satisfying accuracy. The derivative risk stratification strategy classified patients into distinct survival subgroups successfully and performed better than the traditional TNM staging.

**Conclusions:**

The nomogram incorporating *EGFR* mutation status could facilitate survival prediction and risk stratification for individual stage III NSCLC, providing information for enhanced immunotherapy decision and future trial design.

## Graphical abstract

The nomogram incorporating *EGFR* mutation status which could facilitate the 3‐ and 5‐year overall survival prediction for individual stage III NSCLC patients treated with definitive chemoradiotherapy. The derivative risk stratification could successfully classify patients into distinct outcome groups and provide information to enhance immunotherapy decision and future trial design.

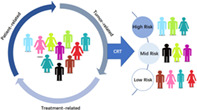

## INTRODUCTION

Lung cancer is the leading cause of cancer death worldwide, with non‐small cell lung cancer (NSCLC) accounting for 85% of all cases.^1^ Approximately one third of patients with NSCLC have locally advanced disease at initial diagnosis.^2^ Definitive concurrent chemoradiotherapy (CRT) has been the backbone therapy for unresectable and medically inoperable stage III NSCLC and 15%–32% patients receiving CRT have been reported to survive at 5 years.^3^, ^4^, ^5^ Recently, the PACIFIC trial demonstrated durvalumab (Imfinzi, AstraZeneca) as consolidation therapy significantly improved the survival of patients who had no progression after CRT with 5‐year overall survival (OS) of 42.9%.^6^, ^7^ Durvalumab was then licensed and became the new standard of care for patients in this disease setting.^8^

Due to the prominent heterogeneity of locally advanced NSCLC (LANSCLC), survival of patients varied widely and whether all the patients were suitable for consolidated immunotherapy remained unclear.^9^, ^10^, ^11^ Therefore, predicting survival and identifying patients at low or high risk of death after CRT were essential for individualized treatment and enhanced immunotherapy decisions. The American Joint Committee on Cancer (AJCC) TNM staging system was the gold standard for the survival risk classification, but was initially developed to evaluate operability rather than outcome after CRT. For prediction and risk stratification in LANSCLC patients, the solely TNM‐based method might be more inaccurate. It was previously reported that other factors such as sex, histology and hematological indicators significantly impact on individual survival.^12^, ^13^, ^14^ Also, the prognostic value of epidermal growth factor receptor (*EGFR*) mutations in adenocarcinoma was increasingly being understood, which led to further molecular heterogeneity.^15^

Therefore, a dedicated prediction model integrating multiple factors for unresectable or inoperable stage III NSCLC patients was urgently needed. A nomogram has been acknowledged as a reliable tool with multivariate visualization to predict the prognosis of patients with malignancies.^16^, ^17^ To date, limited attempts to develop prognostic models for LANSCLC have been reported.^14^, ^18^ In this study, we aimed to build and validate a new nomogram incorporating clinical, treatment‐related and molecular features of *EGFR* mutation to predict the 3‐ and 5‐year OS, by exploring prognostic factors in a large population of LANSCLC patients treated with CRT. An independent cohort from the prospective clinical trial (NCT01494558) was used for external validation.^19^ In addition, based on the model, the cutoff values were determined to stratify patients into different risk subgroups according to the outcome.

## METHODS

### Study cohort

This study was conducted with the approval of our institutional review board. Consecutive patients who received definitive CRT in our institution between January 1st, 2013 and December 31st, 2017 were retrospectively reviewed. As in the PACIFIC trial, consolidative durvalumab was administered for unresectable, stage III NSCLC patients without disease progression after concurrent CRT and the ongoing new series of trials also enrolled patients receiving sequential CRT.^6^, ^7^ The inclusion criteria were designed as follows: (1) patients aged 18 years or older, (2) initially diagnosed with stage III NSCLC by pathology and radiography, (3) unresectable or medically inoperable, (4) received concurrent or sequential chemotherapy, (5) completed a total radiation dose ≥50 Gy with intensity‐modulated radiotherapy (IMRT) technique, and (6) received regular follow‐up with thoracic and abdominal computed tomography (CT), brain magnetic resonance imaging (MRI) and bone emission computed tomography (ECT) or positron emission‐computed tomography (PET). The exclusion criteria included: (1) patients who progressed or died during chemoradiotherapy, (2) were diagnosed with a second primary cancer, and (3) had incomplete clinical information. Tumor staging was evaluated according to the AJCC eighth edition TNM classification and staging system by two investigators retrospectively.

The patients included in the study were randomly stratified (2:1) into the training and testing groups. To examine the generalizability of the model, an independent external cohort from a prospective, randomized phase III trial (NCT01494558) was used for validation. Participants from this trial were diagnosed as unresectable or inoperable stage III NSCLC and treated with definitive CRT (thoracic radiotherapy of 60–66 Gy and platinum‐based chemotherapy)^19^ and only patients meeting the inclusion criteria and with sufficient clinical data to score all factors in the established nomogram were included.

### Data collection

Medical records were reviewed to obtain patient, tumor and treatment‐related information and a standardized data form including all the factors was created to collect the data. Continuous factors were listed with the median and range, whereas categorical factors were summarized by the frequency and proportion. Patient‐related factors included: sex, age, Karnofsky performance status (KPS) score, smoking history, pretreatment peripheral hematological indicators as neutrophil‐to‐lymphocyte ratio (NLR), platelet‐to‐lymphocyte ratio (PLR) and systemic immune‐inflammation index (SII, calculated as platelet counts × neutrophil counts/lymphocyte counts). Tumor‐related factors contained histology (including *EGFR* mutation status in nonsquamous NSCLC), tumor size (maximum diameter), clinical TNM stage, laterality and location (evaluated based on the lobe of the lungs). Regarding treatment‐related factors, gross tumor volume (GTV), planning target volume (PTV), radiation dose, sequence of CRT, chemotherapy regimen and radiation pneumonitis (RP) grade were entailed.

According to treatment strategy, the GTV of radiotherapy (RT) included the primary disease as well as any involved regional lymph nodes, which were defined as those with a short‐axis diameter of at least 1 cm on the CT scan, or with high fluorodeoxyglucose (FDG) uptake on PET‐CT scan. The clinical target volume (CTV) was generated by expanding the GTV with 0.6–0.8 cm, as well as ipsilateral hilum and mediastinal nodal stations involved. The PTV was created by a uniform 0.5 cm expansion around the CTV. The median prescribed dose to PTV was 60 Gy in 30 fractions and ranged from 50 to 72 Gy in 25 to 35 fractions, median biologically equivalent dose (alpha/beta ratio 10 Gy, BED10) of which was 72 Gy. RT was given with 6‐MV X‐rays by linear accelerators and all patients received conventionally fractionated radiotherapy with one fraction per day and five fractions per week. Weekly cone beam computed tomography (CBCT) was acquired for registration throughout the course of radiotherapy. Chemotherapy of platinum‐based double agents was administered every 3 weeks and the dominant regimens included etoposide, paclitaxel or pemetrexed combined with cisplatin or carboplatin. Follow‐up data were collected by the medical records and imaging examinations as previously described. Telephone calls, medical insurance records and death certificates were also required. Overall survival was defined as the time from the date of primary treatment to the date of death.

### Model construction and validation

Cox regression analyses were applied to select prognostic factors in the training group. Variables achieving *p*‐values less than 0.1 by univariate analyses were entered into the multivariate analyses. The final model factors were selected using a backward stepdown process, with the Akaike information criterion as a stopping rule. Based on the results of multivariate analyses, the nomogram was created with Cox proportional hazards model to give the 3‐ and 5‐year OS.

The evaluation of the nomogram comprised the assessment of discrimination and accuracy. Discrimination was calculated with a concordance index (C‐index). The C‐index value of 0.5 indicated a random probability and 1.0 indicated a perfect ability to discriminate outcome. Model accuracy was assessed by the calibration plot. The calibration slope and intercept could measure the agreement between predicted and observed outcomes and a perfect calibration plot would show a 45 upwards line. The internal validation was carried out in the training group with bootstrap resampling (1000 resamples) used. The external validation was implemented in the testing set and the external testing cohort from the prospective trial (NCT01494558). Cox regression analysis, conducted using each patient's total score as an independent factor, was used to evaluate the C‐index and calibration plots. Comparisons between the model and the eighth edition AJCC TNM staging system were performed with integrated discrimination improvement (IDI) to quantify the difference on performance.^20^

### Risk group stratification

In addition to comparing the C‐index numerically, we sought to examine the risk discrimination ability of the model beyond traditional AJCC‐TNM staging. By the X‐tile analysis (Yale University, New Haven, CT, USA) on the model total scores of patients in the training group (from the highest to the lowest), cutoff values were determined to classify the patients into different risk groups.^21^ The cutoffs were then adopted to the testing group and external testing cohort. The Kaplan–Meier survival curves stratified by the risk level and TNM staging were delineated respectively.

### Statistical analysis

Comparisons of the baseline parameters between the training and testing groups were conducted by Chi‐square test or Mann–Whitney U test. Survival curves were estimated with the Kaplan–Meier method and compared with a log‐rank test. All tests were two‐sided, and *p* < 0.05 was defined as a statistically significant result. Statistical analysis was performed by SPSS software (version 25.0) and R (version 4.0.4) via R Studio software (version 1.4.1106). R packages “survival”, “time‐ROC”, “rms”, and “shiny” were used. This study followed the TRIPOD statement.^22^

## RESULTS

### Patient characteristics and survival

A total of 758 LANSCLC patients were treated with CRT from January 1st, 2013 to December 31st, 2017 in our institution and 533 patients were ultimately eligible for analysis based on the inclusion and exclusion criteria. In the whole population, there were 91 (17.1%) females and 442 (82.9%) males with the median age of 60 (range: 23–81). The majority of patients were smoker (76.9%) and had high performance score of KPS ≥ 80 (97.4%). Concerning histology, squamous cell carcinoma (SCC) was diagnosed in 324 (60.8%) patients and among 184 (34.5%) nonsquamous NSCLC patients, 38 (20.7%) patients carried mutant *EGFR*. The median tumor size was 4.4 (range: 0.9–13.4) cm. A total of 127 (23.8%) patients were classified as IIIA stage, whilst 121 (22.7%) were with the IIIC disease. Stratified by a 2:1 ratio, 356 patients were assigned to the training group and 177 to the testing group. The baseline characteristics of patients in the training and testing groups are shown in Table 1. Apart from PLR, no factor presented significant difference between the two groups (*p* > 0.05).

**TABLE 1:** The included characteristics of the training and testing sets

Characteristic *n* (%)	Training set (*n* = 356)	Testing set (*n* = 177)	*p*‐value
Patient characteristics			
Sex			0.302
Male	291 (81.7)	151 (85.3)	
Female	65 (18.3)	26 (14.7)	
Age (median, year)	60 (23–81)	60 (24–77)	0.415
KPS			0.166
70	10 (2.8)	4 (2.3)	
≥80	346 (97.2)	173 (97.7)	
Smoking history			0.290
Non‐smoker	87 (24.4)	36 (20.3)	
Smoker	269 (75.6)	141 (79.7)	
NLR (median)	2.2 (0.4–14.9)	2.3 (0.3–41.6)	0.949
PLR (median)	124.5 (26.6–377.4)	121.4 (25.3–937.8)	0.018
SII (median)	507.6 (56.7–5735.0)	534.2 (24.5–14452.1)	0.757
Tumor characteristics			
Histology			0.580
SCC	215 (60.4)	109 (61.6)	
NS *EGFR* mut−	54 (15.2)	28 (15.8)	
NS *EGFR* mut+	27 (7.6)	11 (3.1)	
NS *EGFR* unknown	40 (11.2)	24 (6.7)	
NOS	20 (5.6)	5 (2.8)	
T stage			0.408
T1	35 (9.9)	20 (11.3)	
T2	99 (27.8)	41 (23.2)	
T3	77 (21.6)	48 (27.1)	
T4	145 (40.7)	68 (38.4)	
N stage			0.868
N0	8 (2.2)	3 (1.7)	
N1	26 (7.4)	10 (5.6)	
N2	166 (46.6)	84 (47.5)	
N3	156 (43.8)	80 (45.2)	
TNM stage			0.111
IIIA	84 (23.6)	43 (24.3)	
IIIB	200 (56.2)	85 (48.0)	
IIIC	72 (20.2)	49 (27.7)	
Laterality			0.763
Left	152 (42.7)	78 (44.1)	
Right	204 (57.3)	99 (55.9)	
Location			0.766
Upper/middle lobe	245 (68.8)	117 (66.1)	
Lower lobe	100 (28.1)	53 (29.9)	
Undefined	11 (3.1)	7 (4.0)	
Tumor size (median, cm)	4.4 (0.9–13.4)	4.5 (1.0–10.5)	0.681
Treatment characteristics			
GTV (ml)	80.7 (3.3–640.5)	69.2 (3.41–668.3)	0.190
PTV (ml)	429.6 (17.1–1195.3)	450.6 (51.4–1317.1)	0.550
RT dose (median, Gy)	60.0 (50.0–72.0)	60 (50.0–70.0)	0.515
CT sequence			0.213
Sequential	161 (45.2)	70 (39.5)	
Concurrent	195 (54.8)	107 (60.5)	
CT regimen			0.358
Etoposide‐platinum	226 (63.5)	108 (61.0)	
Paclitaxel‐platinum	98 (27.5)	46 (26.0)	
Pemetrexed‐platinum‐	32 (9.0)	23 (13.0)	
Radiation pneumonitis			0.240
≤2 grade	332 (93.3)	170 (96.0)	
>2 grade	24 (6.7)	7 (4.0)	

Abbreviations: CT, chemotherapy; GTV, gross tumor volume; KPS, Karnofsky performance score; Mut, mutation; NLR, neutrophil‐to‐lymphocyte ratio; NOS, not otherwise specified; NS, nonsquamous; PLR, platelet‐to‐lymphocyte ratio; PTV, planning target volume; RT, radiotherapy; SII, systemic immune‐inflammation index; SCC, squamous cell carcinoma.

All 533 patients included in the study had survival data and the Kaplan–Meier curve of the overall population was shown in Supplementary Figure S1. There were 298 events (deaths) over a median follow‐up time of 39.6 (range: 4.9–80.8) months and the median survival was 30.6 (95% CI: 26.6–34.6) months. The 3‐ and 5‐year OS for the enrolled patients was 44.2% and 29.6%, respectively.

### Independent prognostic factors

According to the univariate analysis of training group, factors such as female (vs. male, *p* < 0.001), KPS ≥ 80 (vs. 70, *p* = 0.008) and non‐smoker (vs. smoker, *p* = 0.011) were associated with better prognosis. Among all the histological types, nonsquamous NSCLC with mutant *EGFR* showed the survival superiority, followed by NOS, nonsquamous NSCLC without *EGFR* mutations and SCC. Clinical T and N component stage presented no significant correlation with OS with *p* value > 0.05, but the overall clinical TNM stage was an independent factors influencing OS, for patients diagnosed as IIIC stage had significant shorter survival in comparison with IIIA stage (HR = 1.642, 95% CI: 1.090–2.475, *p* = 0.018). In addition, metrical tumor size was a significant parameter for OS (*p* = 0.007), whilst tumor laterality and location were excluded (*p* > 0.05). With respect to treatment‐related factors, PTV volume, RT dose, RP grade and chemotherapy sequence were associated with OS with *p*‐values of <0.001, 0.044, 0.017 and <0.001, yet all pretreatment hematological inflammatory indices demonstrated no statistically significant correlation (*p* > 0.05).

All factors with *p* < 0.1 in univariate analyses were entered into Cox multivariate analyses. Sex, histology, PTV volume, chemotherapy sequence and RP grade retained independent significant factors in the multivariate analyses. The results of univariate and multivariate analyses for OS are listed in Table 2. The Kaplan–Meier curves stratified by these factors and the corresponding *p*‐values are presented in Supplementary Figure S2.

**TABLE 2:** Univariate and multivariate analyses of the included characteristics for overall survival

Characteristic	Univariate analysis		Multivariate analysis	
	HR (95% CI)	*p*‐value	HR (95% CI)	*p*‐value
Patient characteristics				
Sex				
Male	1	‐	1	‐
Female	0.460 (0.305–0.695)	<0.001	0.451 (0.282–0.722)	0.001
Age (median, year)	1.008 (0.992–1.024)	0.351		
KPS				
70	1	‐	1	‐
≥80	0.356 (0.167–0.761)	0.008	1.866 (0.816–4.271)	0.139
Smoking history				
Non‐smoker	1	‐	1	‐
Smoker	1.589 (1.111–2.271)	0.011	1.196 (0.803–1.781)0.	0.380
NLR	1.044 (0.968–1.125)	0.263		
PLR	1.001 (0.998–1.003)	0.661		
SII	1.000 (1.000–1.000)	0.148		
Tumor characteristics				
Histology				
SCC	1	‐	1	‐
NS *EGFR* mut−	0.562 (0.364–0.867)	0.009	0.618 (0.396–0.963)	0.034
NS *EGFR* mut+	0.298 (0.139–0.637)	0.002	0.371 (0.172–0.800)	0.011
NS *EGFR* unknown	1.115 (0.733–1.695)	0.611	1.582 (0.973–2.571)	0.064
NOS	0.479 (0.235–0.979)	0.044	0.598 (0.290–1.236)	0.165
T stage				
T1	1	‐		
T2	0.908 (0.530–1.558)	0.727		
T3	1.343 (0.775–2.326)	0.293		
T4	1.360 (0.821–2.253)	0.232		
N stage				
N0	1	‐		
N1	0.772 (0.281–2.126)	0.617		
N2	1.035 (0.421–2.544)	0.940		
N3	0.970 (0.394–2.390)	0.947		
TNM stage				
IIIA	1	‐	1	‐
IIIB	1.105 (0.784–1.557)	0.569	1.059 (0.718–1.562)	0.774
IIIC	1.642 (1.090–2.475)	0.018	1.569 (0.989–2.489)	0.056
Laterality				
Left	1	‐		
Right	0.915 (0.694–1.206)	0.528		
Location				
Upper/middle lobe	1	‐		
Lower lobe	1.161 (0.853–1.580)	0.342		
Undefined	1.107 (0.489–2.509)	0.807		
Tumor size	1.097 (1.026–1.174)	0.007	1.020 (0.942–1.104)	0.632
Treatment characteristics				
GTV	1.000 (0.999–1.002)	0.814		
PTV	1.002 (1.001–1.002)	<0.001	1.001 (1.001–1.002)	0.001
RT dose	0.956 (0.915–0.999)	0.044	0.955 (0.910–1.002)	0.062
CT sequence				
Sequential	1	‐	1	‐
Concurrent	0.714 (0.541–0.941)	0.017	0.594 (0.436–0.809)	0.001
CT regimen				
Etoposide‐platinum	1	‐		
Paclitaxel‐platinum	1.390 (0.896–2.157)	0.142		
Pemetrexed‐platinum‐	0.532 (0.195–1.456)	0.219		
Radiation pneumonitis				
≤2 grade	1	‐	1	‐
>2 grade	2.798 (1.715–4.563)	<0.001	3.319 (1.989–5.536)	<0.001

Abbreviations: CI, confidence interval; CT, chemotherapy; GTV, gross tumor volume; HR, hazard ratio; KPS, Karnofsky performance score; Mut, mutation; NLR, neutrophil‐to‐lymphocyte ratio; NOS, not otherwise specified; NS, nonsquamous; PLR, platelet‐to‐lymphocyte ratio; PTV, planning target volume; RT, radiotherapy; SII, systemic immune‐inflammation index; SCC, squamous cell carcinoma.

### Nomogram development and validation

**FIGURE 1:**
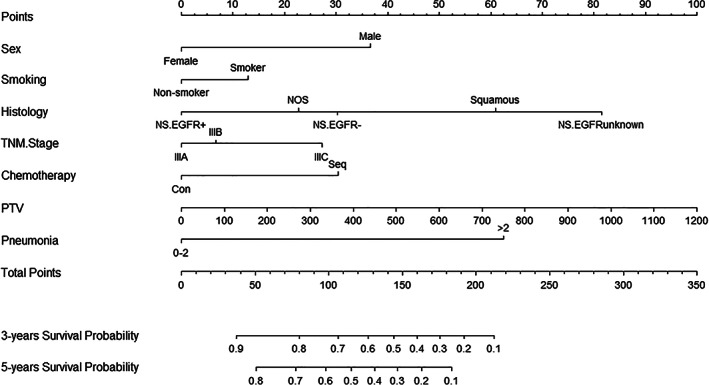
Predictive nomogram for the 3‐ and 5‐year overall survival in stage III NSCLC patients treated with chemoradiotherapy. GTV, gross tumor volume; Mut, mutation; NOS, not otherwise specified; NS, nonsquamous; PTV, planning target volume; SCC, squamous cell carcinoma

The training set was used for model construction and a nomogram was established with all the selected factors incorporated (Figure 1). Based on previous reports and clinical experience, overall TNM stage and smoking history were also involved to the development in order to improve the discriminative ability. By summing the total score of seven variables and locating it on the total points scale, the estimated 3‐ and 5‐year OS could be easily determined.

**FIGURE 2:**
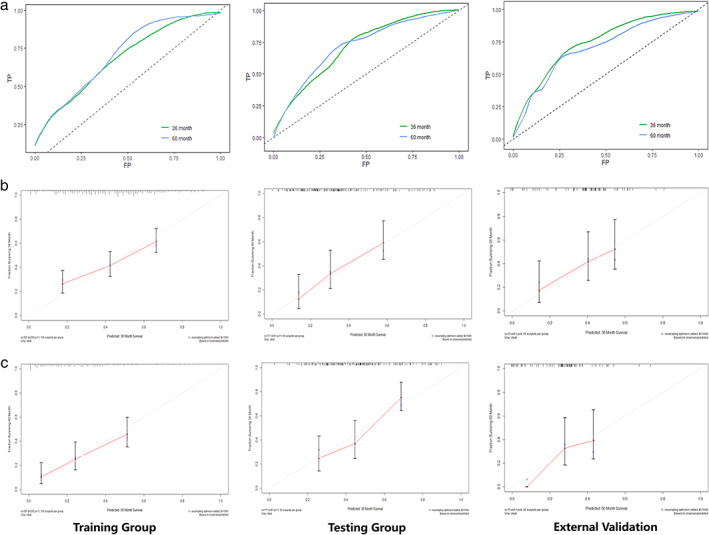
The receiver operating characteristic (ROC) curves and the area under curves (AUC) of the predictive nomogram (a); the calibration curves for predicting (b) the 3‐year and (c) 5‐year overall survival in the training, testing group and external trial cohort. A plot along 45‐degree line indicated the perfect model in which the predicted equaled to the actual survival

Discrimination and accuracy of the nomogram was examined. In the training group, the C‐index was 0.688 (95% CI: 0.633–0.743) for the 3‐year and 0.710 (95% CI: 0.653–0.767) for the 5‐year OS. For validation, the 3‐ and 5‐year C‐indexes were 0.717 (95% CI: 0.639–0.795), 0.720 (95% CI: 0.640–0.800) in the testing group and 0.744 (95% CI: 0.607–0.881), 0.699 (95% CI: 0.550–0.848) in the external testing cohort, respectively (Figure 2a). In all the three cohorts, the calibration plots presented excellent accordance and acceptable agreement between the nomogram predictions and actual observations for the 3‐ and 5‐year OS (Figure 2b,c).

### Comparison of the model and AJCC TNM staging system

A comparison between the nomogram and the eighth edition AJCC TNM staging system was conducted. Compared with the eighth edition AJCC TNM staging system, the IDI for 3‐ and 5‐year OS of the new model was 12.729% (*p* < 0.001) and 11.504% (*p* < 0.001). In addition, time‐dependent ROC curves for OS showed the new model performed better prediction ability than the classical TNM staging system consistently in the three cohorts (Supplementary Figure S3).

### Risk group stratification

**FIGURE 3:**
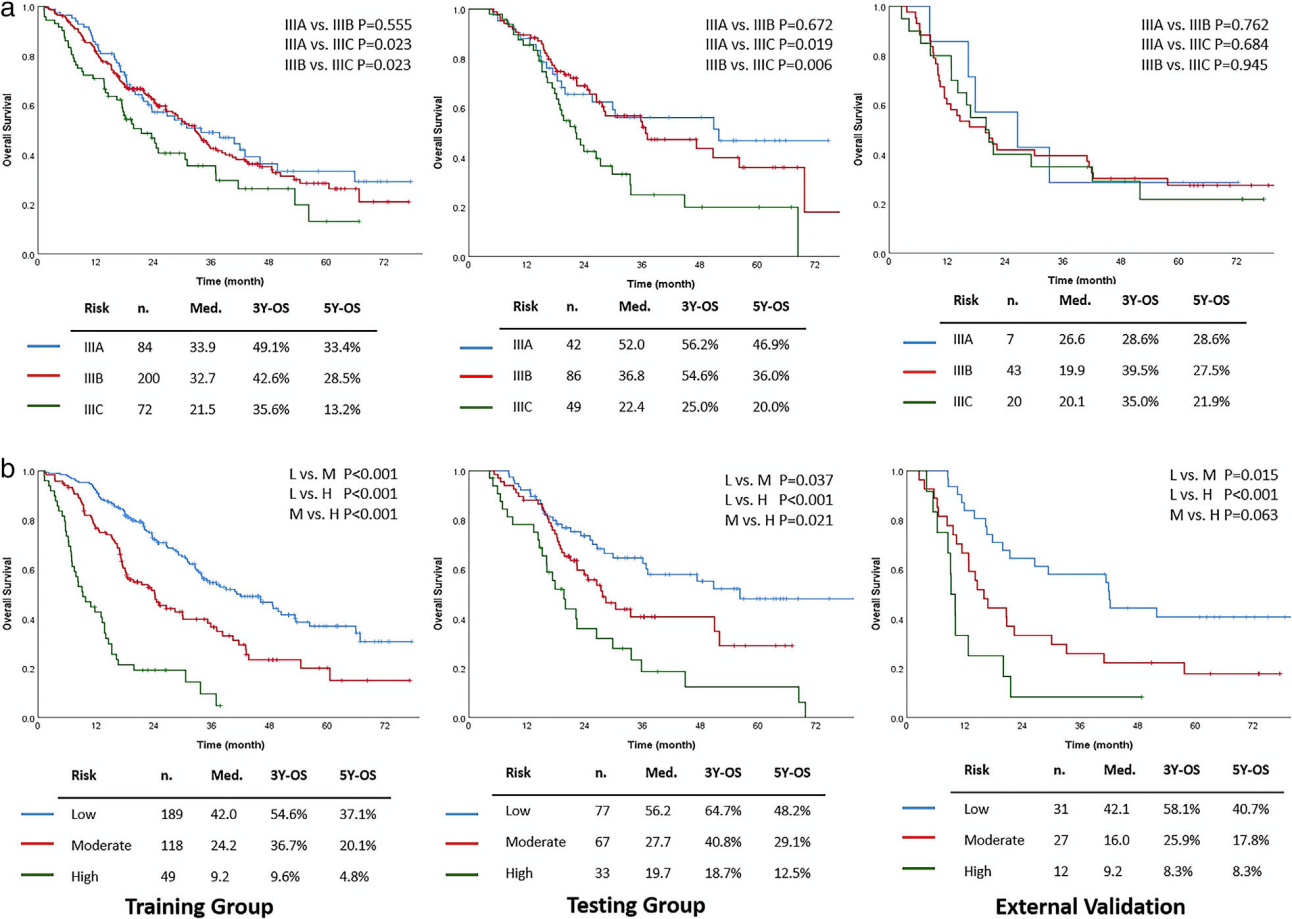
The Kaplan–Meier curves for overall survival of patients stratified by (a) the eighth edition AJCC TNM staging system and (b) the risk stratification strategy derived from the nomogram

We calculated the cutoff values by sorting the total score (TS) in the training set and grouped patients into three subgroups (low‐risk: TS < 160, moderate‐risk: 160 ≤ TS < 200, high‐risk: 200 ≤ TS). The Kaplan–Meier survival curves demonstrated distinct prognosis of each subgroup. The cutoff values were then applied to stratify patients in the testing set and the external validation cohort, which also presented significant differences between various risk subgroups (Figure 3b). In contrast, the eighth edition TNM staging showed inferior capacity of classification with the insignificant survival difference between patients with IIIA and IIIB stage (Figure 3a).

### Easy access of the new model

For convenience of use, the online version of our nomogram can be accessed at https://la-nsclc-crt.shinyapps.io/LANSCLC-Prediction/. By inputting model variables, predicted survival probability can easily be determined with the figures and tables generated by the website (Supplementary Figure S4). In addition, we further simplified the model as a scoring system to assist researchers and clinicians to evaluate prognosis and stratify cohorts (Supplementary Table S1).

## DISCUSSION

Survival prediction for stage III NSCLC treated with CRT is quite challenging. A nomogram has been acknowledged as a more accurate tool of predicting prognosis than the TNM classification for NSCLC.^17^, ^23^, ^24^ Since the nomograms for early‐stage NSCLC are well established, existing nomograms concerning LANSCLC are scarce and still need adjustment and improvement. Initially in 2015, Oberije et al. established a predictive model of 2‐year OS for stage III NSCLC with RT or CRT, yielding the moderate C‐statistics of 0.65 in internal validation and 0.58, 0.60 in the external data sets.^14^ The results were reasonable as it must be difficult to predict and discriminate prognosis for a recognized heterogeneous subgroup of inoperable patients within the same clinical stage. Recently, Wang et al. proposed a second nomogram for LANSCLC incorporating clinical and radiomic features, which obtained a satisfying C‐index of 0.74 by cross validation when evaluating the 2‐year survival. However, the study was limited by its small sample size of 118 patients and no independent external validation.^18^

In this study, we developed and validated a new nomogram to predict the long‐term survival of the 3‐ and 5‐year OS for unresectable or medically inoperable stage III NSCLC patients with definitive CRT. By utilizing patient, tumor and treatment‐related factors which are all generally documented by oncologists for NSCLC patients, our model improved upon the two prior nomograms in the training sample size, predictive ability and application potential in the clinical practice. In addition, external validation was conducted in both the testing group and a prospective trial cohort. As a result, the model showed evidently better predictive capacity than the TNM classification.

LANSCLC is acknowledged as a highly heterogeneous stage of disease with diverse tumor burden and clinical factors, and several clinical features have emerged to affect the survival.^25^, ^26^, ^27^ Based on previous reports and our experience, patient, tumor and treatment‐related factors were brought into the univariate and multivariate analyses, and ultimately seven parameters of sex, smoking history, histology (including *EGFR* mutation status), overall TNM stage, chemotherapy sequence, PTV volume and RP grade were determined as important prognostic factors.

These findings are in concordance with previous observations. In addition to advanced TNM classification, male gender and smoking history are constantly reported to be associated with a high risk of death.^27^ With regard to tumor histology, our model was novel in the incorporation of *EGFR* mutation status in nonsquamous NSCLC, which is a genetic feature identified with prognostic significance. It has been reported that EGFR pathway activation performs a molecular basis of radiation resistance and prognostic value of the specific gene alterations still need further investigation in LANSCLC. Since the genetic profile guided treatment was widely used in metastatic NSCLC, the molecular variations rarely affected primary treatment options for stage III patients yet, thus *EGFR* mutation data was not obtained in 40 (33.1%) patients in the training group. According to current literature, nonsquamous NSCLC patients harboring *EGFR* mutations had longer local control and survival than patients with wild‐type *EGFR* after definitive CRT.^28^, ^29^, ^30^ Our results also demonstrated the survival advantage of *EGFR* mutant nonsquamous patients, but it is worth noting that most *EGFR* mutant patients (86.8%) were recorded to receive *EGFR* tyrosine kinase inhibitors after progression, leading to the possible post‐progression survival benefit of this group of patients. Interestingly, all the hematological indices included such as NLR, PLR and SII presented no significant impact on OS, contradicting the promising prognostic value of these immune‐inflammation indices proposed by prior studies.^31^, ^32^, ^33^, ^34^ However, the fact that the hematological indices were evaluated merely before treatment and analyzed as continuous variables without appropriate cutoff points in this study could be possible reasons for this. The determination of optimal cutoffs and investigations on the dynamic evaluation of the indices were warranted.

With regard to treatment‐related factors, PTV volume, chemotherapy sequence and radiation pneumonitis grade are associated with OS, all of which are supported by previous evidence. It has been proven that increased volume of PTV is related to a higher risk of death and that concurrent CRT was preferred over sequential CRT in large scale clinical trials and meta‐analyses.^2^, ^5^, ^35^, ^36^ The occurrence of severe radiation pneumonitis (grade > 2) has also been revealed as a crucial treatment‐related toxicity predicting poor survival, possibly due to the induced pulmonary fibrosis and chronic respiratory insufficiency.^37^ Consistent with the results of RTOG 0617 and PROCLAIM trial, both the RT dose and chemotherapy regimen were excluded with no significant survival influence.^5^, ^38^, ^39^

Based on Cox proportional hazards analysis, the final model was built integrating the seven significant factors and validated in both the testing and external testing trial cohorts to determine generalizability. The C‐indexes of our model remained stable ranging from 0.688 to 0.744 across the cohorts and calibration plots displayed ideal agreement between the prediction and actual observations, which guaranteed the improved accuracy and reliability of the nomogram. In comparison with TNM classification, IDI of 12.729% and 11.504% showed the superior performance of our model to predict 3‐ and 5‐year survival. Our results also suggested that TNM staging was not sufficient to divide patients into distinct risk groups consistently, especially in the less‐risky patients of IIIA and IIIB stages, but the new nomogram could separate patients with different outcomes successfully in all the groups, indicating it a useful tool for individual risk identification and follow‐up strategy‐making. According to the new categories divided by our nomogram, it should be noted that the median survival of low‐risk subgroup commonly exceeded 42 months, numerically approaching the median of 47.5 months of patients from the durvalumab group in the PACIFIC trial.^6^ From this point, the nomogram would also give reference for enhanced immunotherapy decision and future trial design with better equivalence between arms.

To our knowledge, this nomogram is the first model to predict long‐term 3‐ and 5‐year individual survival, combining tumor *EGFR* mutation status and treatment‐related factors in stage III NSCLC patients with CRT. Comparative information of the present prediction models for LANSCLC is summarized in Table 3.^14^, ^18^, ^40^ This quantitative multivariate model obtained distinctively better predictive ability by comprehensive evaluation and validation. The incorporations of the new molecular factors above were considered responsible for the improvement in performance. The easy‐to‐obtain clinical variables and easy‐to‐use website/simplified scoring system equipped the model with high value of practical utility.

**TABLE 3:** Summary of the current prediction models for overall survival in stage III NSCLC treated with definitive chemotherapy

Study (year)	Sample size (n.)	Predict endpoint	Prognostic factors	C‐statistics/C‐indexes	External validation
Oberije et al. 2015^14^	548	2‐year OS	Age, gender, WHO‐PS, GTV, PLNS (PET), T stage, EQD2, OTT	0.59–0.62	Yes
Wang et al. 2019^18^	118	1‐ and 2‐year OS	Age, lymph node, lymphocyte, NLR, radiomics signature	0.743	No
Chen et al. 2020^40^	227	3‐year OS	TNM stage, GTV	0.636	Yes
This study	356	3‐ and 5‐year OS	Sex, smoking history, histology (*EGFR* status included), TNM stage, chemotherapy sequence, PTV, radiation pneumonitis	0.688–0.744	Yes

Abbreviations: EQD2, equivalent dose in 2 Gy fractions; GTV, gross tumor volume; NLR, neutrophil‐to‐lymphocyte ratio; OS, overall survival; OTT, overall treatment time; PLNS (PET), positive lymph node stations (on positron emission tomography); PTV, planning target volume; WHO‐PS, World Health Organization performance status.

However, there were several limitations of the present study. First, the nomogram and cutoff values for risk stratification were developed within a single‐institution retrospective database. Although validated in a prospective trial cohort externally, the model still needs further evaluation by larger scale multicenter data to reduce the bias. Second, the model failed to incorporate several recognized prognostic parameters such as tumor standard uptake value (SUV) measurements on PET‐CT and immunohistochemical indicators (e.g., PD‐L1 expression and CD8+ tumor infiltrating lymphocytes).^41^, ^42^, ^43^, ^44^ Nevertheless, the assessments of some quantitative factors were hard to standardize across different years and clinical practice, confining the extensive application of these parameters. Third, apart from *EGFR* mutation, other crucial molecular markers (e.g., *KRAS* mutation and *ALK* rearrangement) also showed prognostic potential in LANSCLC, which were not integrated in the current model.^45^, ^46^ The development and widespread use of genomic and proteomics testing are of great promise to realize more accurate prediction. Therefore, efforts on collection of multicenter data and incorporation of comprehensive refined factors are encouraged to optimize this model in the future.

In conclusion, we established and validated a new nomogram incorporating molecular features of *EGFR* mutations to predict the 3‐ and 5‐year OS for LANSCLC patients receiving definitive CRT. This model will facilitate the accurate survival prediction and risk stratification of individual patients, providing information to enhance immunotherapy decision‐making and future clinical trial design.

## CONFLICT OF INTEREST

None of the authors of this study had any relevant conflicts of interest.

## Supporting information


**Figure S1**. The Kaplan–Meier curve for overall survival of all the included 533 patients.
**Figure S2**. The Kaplan–Meier curves stratified by the important predictive factors (except the continuous variable of PTV volume).
**Figure S3**. The time‐dependent receiver operating characteristic (ROC) curves for overall survival comparing the 8th edition AJCC TNM staging system and the nomogram.
**Figure S4**. The easy‐to‐use website of survival prediction for stage III NSCLC treated definitive chemoradiotherapy.
**Table S1**. The simplified scoring system derived from the new nomogram. Abbreviations: NS = nonsquamous, Mut = mutation, NOS = not otherwise specified, SCC = squamous cell carcinoma, PTV = planning target volume.
