# Supplementary material for: Development and validation of a prediction model using molecular marker for long‐term survival in unresectable stage III non‐small cell lung cancer treated with chemoradiotherapy
Source: Thorac Cancer. 2021 Dec 19;13(3):296–307. doi: 10.1111/1759-7714.14218 (PMC8807329; doi:10.1111/1759-7714.14218)
Supplement: Supplementary file 1 — Figure S1. The Kaplan–Meier curve for overall survival of all the included 533 patients. Figure S2. The Kaplan–Meier curves stratified by the important predictive factors (except the continuous variable of PTV volume). Figure S3. The time‐dependent receiver operating characteristic (ROC) curves for overall survival comparing the 8th edition AJCC TNM staging system and the nomogram. Figure S4. The easy‐to‐use website of survival prediction for stage III NSCLC treated definitive chemoradiotherapy. Table S1. The simplified scoring system derived from the new nomogram. Abbreviations: NS = nonsquamous, Mut = mutation, NOS = not otherwise specified, SCC = squamous cell carcinoma, PTV = planning target volume. [file TCA-13-296-s001.docx]

**SUPPLEMENTS**

Supplementary Figure 1.

The Kaplan-Meier curve for overall survival of all the included 533 patients. **(Page 2)**

Supplementary Figure 2.

The Kaplan-Meier curves stratified by the important predictive factors (except the continuous variable of PTV volume). **(Page 3)**

Supplementary Figure 3.

The time-dependent receiver operating characteristic (ROC) curves for overall survival comparing the 8th edition AJCC TNM staging system and the nomogram. **(Page 4)**

Supplementary Figure 4.

The easy-to-use website of survival prediction for stage III NSCLC treated definitive chemoradiotherapy. **(Page 5)**

Supplementary Table 1.

The simplified scoring system derived from the new nomogram. **(Page 6)**

Abbreviations: NS = nonsquamous, Mut = mutation, NOS = not otherwise specified, SCC = squamous cell carcinoma, PTV = planning target volume.

Supplementary Figure 1.

The Kaplan-Meier curve for overall survival of all the included 533 patients.


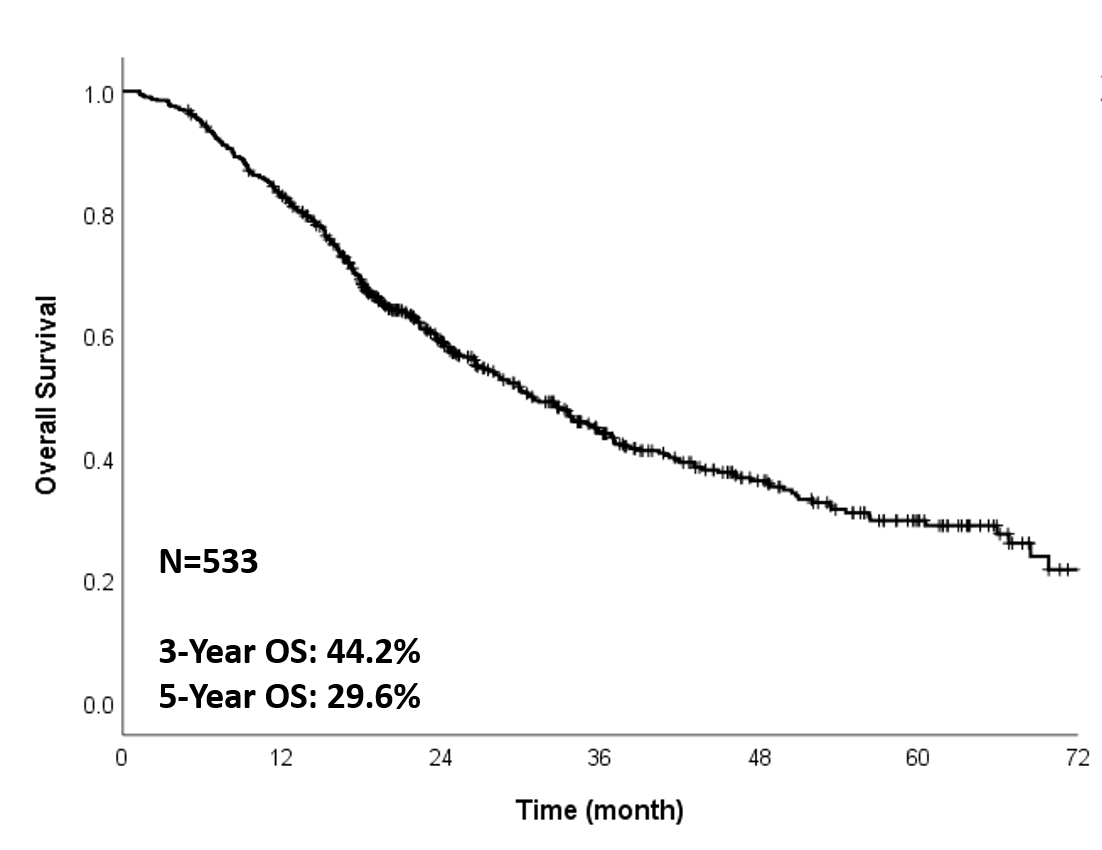


Supplementary Figure 2.

The Kaplan-Meier curves stratified by the important predictive factors (except the continuous variable of PTV volume).


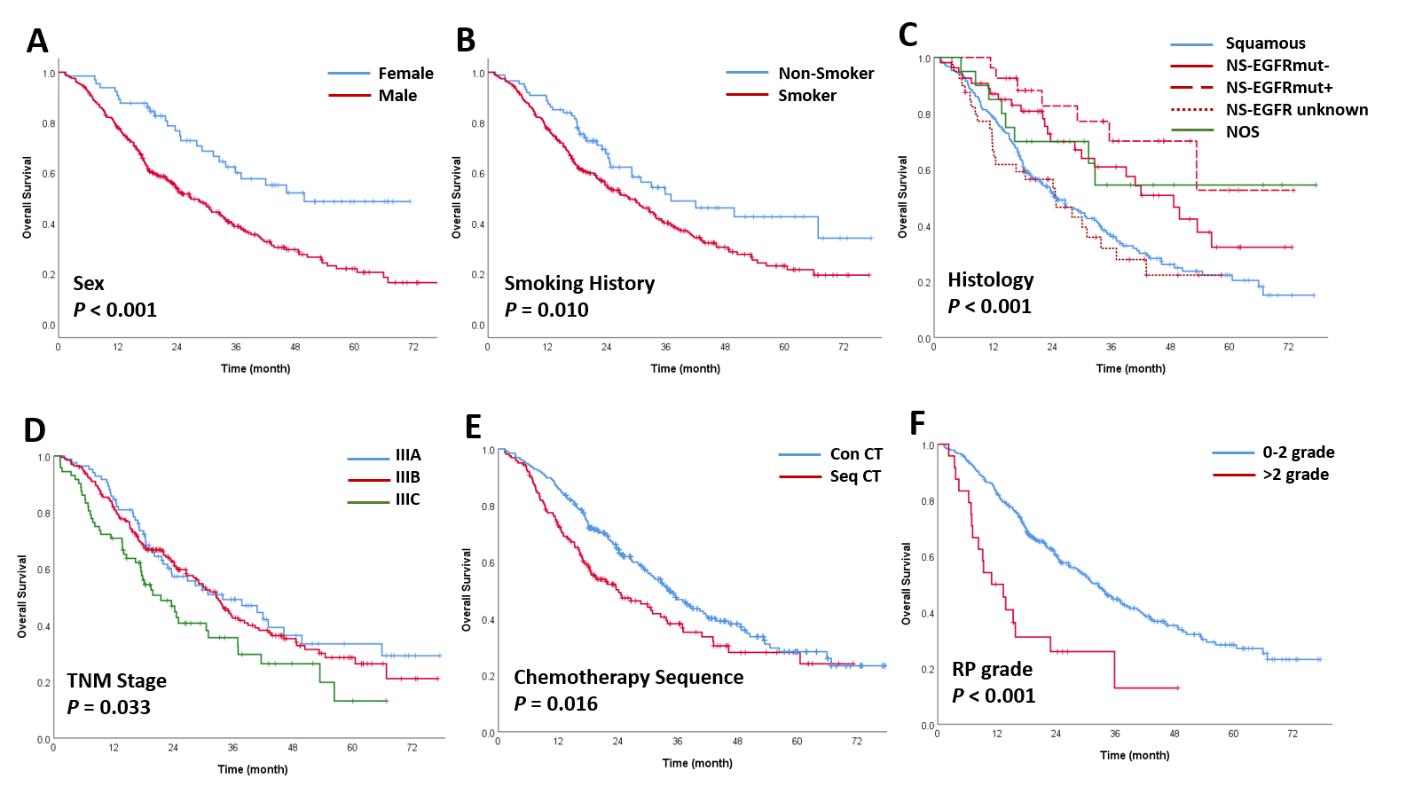


Supplementary Figure 3.

The time-dependent receiver operating characteristic (ROC) curves for overall survival comparing the 8th edition AJCC TNM staging system and the nomogram.


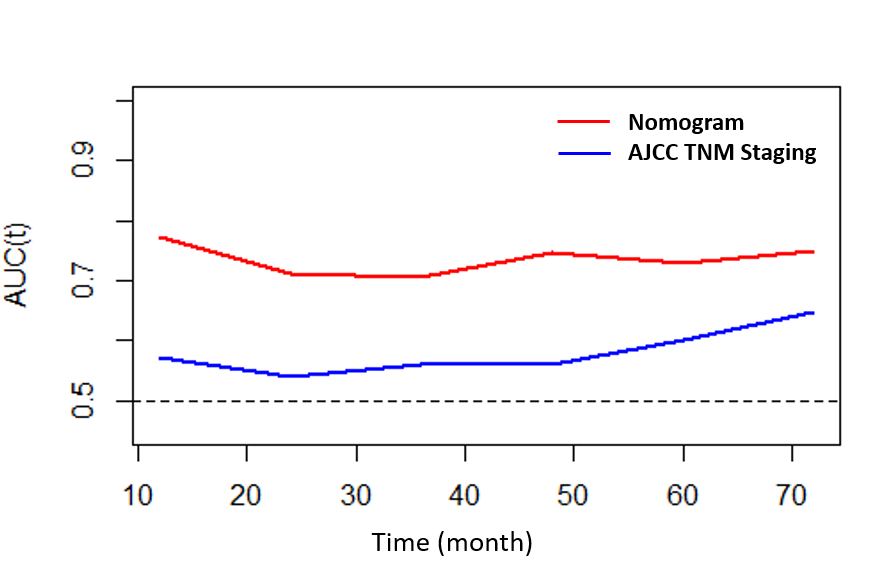


Supplementary Figure 4.

The easy-to-use website of survival prediction for stage III NSCLC treated definitive chemoradiotherapy.


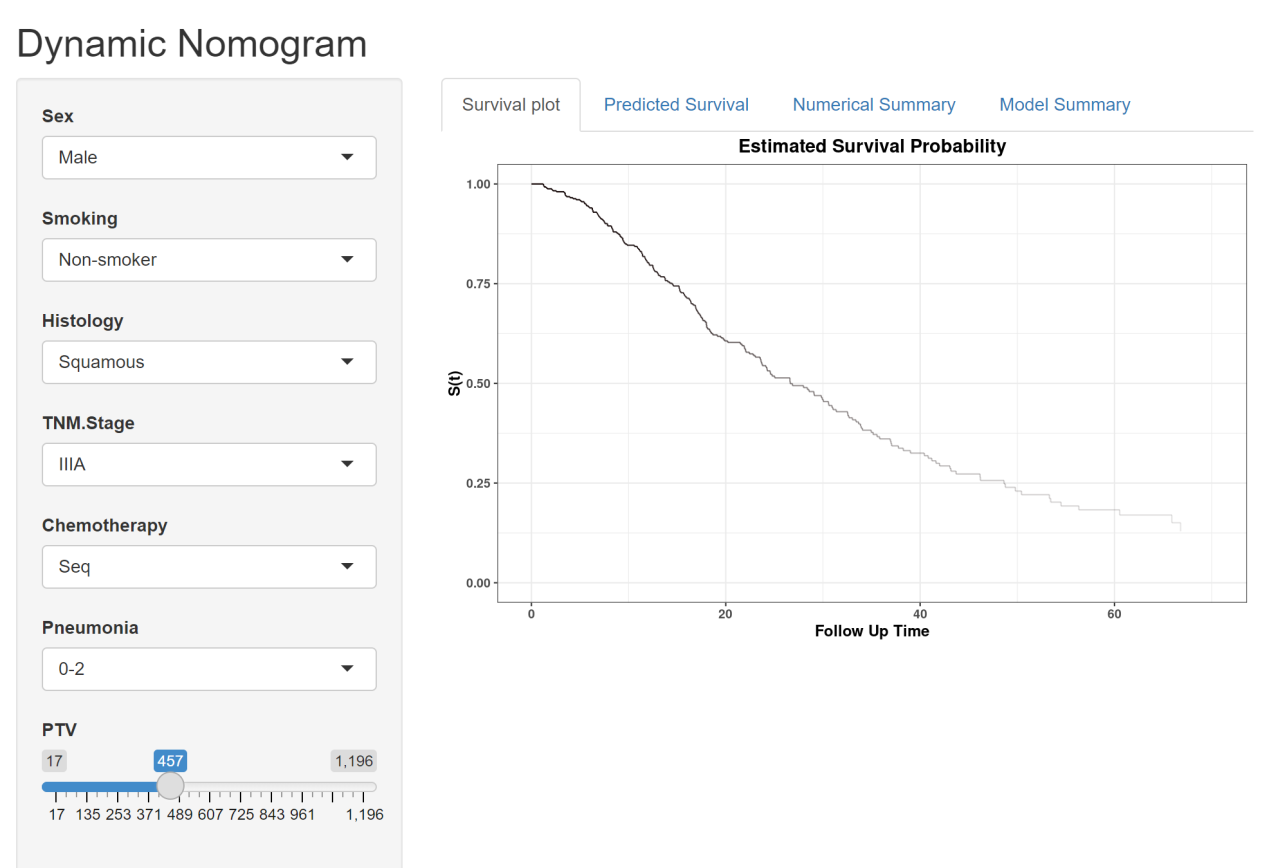


Supplementary Table 1.

The simplified scoring system derived from the new nomogram.

Abbreviations: NS = nonsquamous, Mut = mutation, NOS = not otherwise specified, SCC = squamous cell carcinoma, PTV = planning target volume.

| Characteristic | Point |
| --- | --- |
| Sex |  |
| Female | 0 |
| Male | 4 |
| Smoking History |  |
| Non-Smoker | 0 |
| Smoker | 1 |
| Histology |  |
| NS EGFR mut+ | 0 |
| NOS | 2 |
| NS EGFR mut- | 3 |
| SCC | 6 |
| NS EGFR unknown | 8 |
| TNM Stage |  |
| IIIA | 0 |
| IIIB | 1 |
| IIIC | 3 |
| Chemotherapy Sequence |  |
| Concurrent | 0 |
| Sequential | 3 |
| Radiation Pneumonitis |  |
| ≤2 Grade | 0 |
| >2 Grade | 6 |
| PTV (ml) | PTV*0.008 |
| Risk Group |  |
| Low | 0≤ Total <16 |
| Moderate | 16≤ Total <20 |
| High | Total ≥20 |
